# New Power Sharing Control for Inverter-Dominated Microgrid Based on Impedance Match Concept

**DOI:** 10.1155/2013/816525

**Published:** 2013-12-23

**Authors:** Herong Gu, Deyu Wang, Hong Shen, Wei Zhao, Xiaoqiang Guo

**Affiliations:** Key Lab of Power Electronics for Energy Conservation and Motor Drive of Hebei Province, Yanshan University, Qinhuangdao 066004, China

## Abstract

Power flow control is one of the most important issues for operating the inverter-dominated autonomous microgrid. A technical challenge is how to achieve the accurate active/reactive power sharing of inverters. *P*-*F* and *Q*-*V* droop control schemes have been widely used for power sharing in the past decades. But they suffer from the poor power sharing in the presence of unequal line impedance. In order to solve the problem, a comprehensive analysis of the power droop control is presented, and a new droop control based on the impedance match concept is proposed in this paper. In addition, the design guidelines of control coefficients and virtual impedance are provided. Finally, the performance evaluation is carried out, and the evaluation results verify the effectiveness of the proposed method.

## 1. Introduction

The environmental concerns and electric utility deregulation promote the development of distributed generation (DG) in a rapid pace. The high penetration of DG brings about a concept of the microgrid. It is defined as a cluster of DG units (such as wind turbines and photovoltaics), storage devices, and loads, which can operate in the grid-connected mode or autonomous mode [1], and this paper focuses on the latter.

For inverter-based autonomous microgrid, the droop control is widely used to regulate the power flow according to the local information with no need of communication [2–6]. Generally speaking, an ideal droop control should provide the fast and accurate power sharing without affecting the voltage and frequency at the point of common coupling (PCC). In practice, however, the widely used droop control, which stems from the original philosophy of the synchronous generator control in power systems, fails to meet the above-mentioned requirements. In the past decades, many attempts have been made to improve the performance of the conventional droop control. A significant contribution from Josep Guerrero is the virtual impedance concept, which demonstrates that the inverter output impedance depends on not only the system parameters but also the inverter control scheme [7]. After that, the virtual impedance based droop control has gained more and more attention. For the accurate power sharing, the output impedance should be fixed as inductive, resistive, or complex impedances. In [8], a virtual inductance is designed for the inductive output impedance even with high *R*/*X* ratio. On the other hand, the resistive output impedance is used [2], which ensures that the system is more damped and achieves better power sharing. In [9], the virtual complex impedance is designed to minimize the circulating current for the efficient power sharing.

On the other hand, an improved droop method has been reported in [10], where the basic idea is to transform the actual power to the virtual power according to *R*/*X* ratio of the line impedance, and then the conventional droop control can be used. Another interesting solution reported in [11] is the virtual frequency and voltage frame droop control. Different from the former control, it can directly control the actual real and reactive power, but the frame transformation angle for each inverter should be the same (e.g., 45°). Note that two aforementioned methods can get better performance from the viewpoint of power decoupling. However, power sharing is still a question, especially in the presence of unequal line impedance.

In this paper, a comprehensive analysis is provided to clarify why the conventional droop control can achieve the accurate active power sharing but fail to get better reactive power sharing. And then a simple and accurate power sharing scheme is presented to solve the problem. Finally, the performance evaluation is carried out to verify the feasibility of the proposed method.

## 2. Analysis and Design of Droop Control

**Figure 1:**
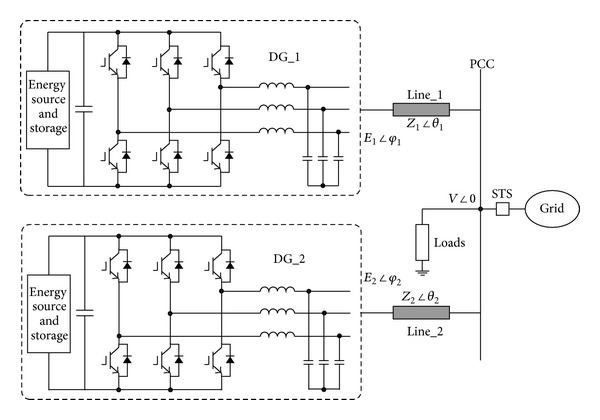
Schematic diagram of inverter-based microgrid.

Figure 1 illustrates the schematic diagram of inverter-based microgrid [11]. It is comprised of the energy sources with optional energy storages and dc/ac inverters. The inverters can provide an interface for the flexible functions such as power flow control and power quality improvement. The inverter output may either feed the local loads independently in autonomous mode or in conjunction with the electric utility by static switch (STS) in grid-connected mode. This paper will focus on the former mode.

### 2.1. Power Droop Theory

The power droop control has a long history of use for the synchronous generator control in power system. Recently, it has been used for parallel-inverter control, especially in inverter-dominated microgrid [12]. The following will provide a brief review of the conventional power droop scheme.

The inverter output active and reactive power can be expressed according to Figure 1 as follows:

(1) $$\begin{array}{c} P_{i}=\frac{R_{i}E_{i}^{2}-R_{i}E_{i}V\cos\varphi_{i}+X_{i}E_{i}V\sin\varphi_{i}}{R_{i}^{2}+X_{i}^{2}},\\ Q_{i}=\frac{X_{i}E_{i}^{2}-X_{i}E_{i}V\cos\varphi_{i}-R_{i}E_{i}V\sin\varphi_{i}}{R_{i}^{2}+X_{i}^{2}}, \end{array}$$

where *E*_*i*_ and *V* are the amplitudes of the *i*th inverter output voltage, and PCC voltage respectively. *φ*_*i*_ is the power angle. *R*_*i*_ and *X*_*i*_ are the resistance and inductance of the line impedance.

In order to clarify the basic principle of the power droop control, the sensitivity analysis is carried out as follows:

(2) $$\begin{array}{c} \frac{\partial P_{i}}{\partial \varphi_{i}}=\frac{R_{i}E_{i}V\sin\varphi_{i}+X_{i}E_{i}V\cos\varphi_{i}}{R_{i}^{2}+X_{i}^{2}},\\ \frac{\partial P_{i}}{\partial E_{i}}=\frac{2R_{i}E_{i}-R_{i}V\cos\varphi_{i}+X_{i}V\sin\varphi_{i}}{R_{i}^{2}+X_{i}^{2}},\\ \frac{\partial Q_{i}}{\partial \varphi_{i}}=\frac{X_{i}E_{i}V\sin\varphi_{i}-R_{i}E_{i}V\cos\varphi_{i}}{R_{i}^{2}+X_{i}^{2}},\\ \frac{\partial Q_{i}}{\partial E_{i}}=\frac{2X_{i}E_{i}-X_{i}V\cos\varphi_{i}-R_{i}V\sin\varphi_{i}}{R_{i}^{2}+X_{i}^{2}}. \end{array}$$

Note that the power angle *φ*_*i*_ is relatively small in practice; that is, sin*φ*_*i*_ ≈ 0 and cos⁡*φ*_*i*_ ≈ 1. Equation (2) can be simplified as

(3) $$\begin{array}{c} \frac{\partial P_{i}}{\partial \varphi_{i}}=\frac{R_{i}E_{i}V\varphi_{i}+X_{i}E_{i}V}{R_{i}^{2}+X_{i}^{2}},\\ \frac{\partial P_{i}}{\partial E_{i}}=\frac{2R_{i}E_{i}-R_{i}V+X_{i}V\varphi_{i}}{R_{i}^{2}+X_{i}^{2}},\\ \frac{\partial Q_{i}}{\partial \varphi_{i}}=\frac{X_{i}E_{i}V\varphi_{i}-R_{i}E_{i}V}{R_{i}^{2}+X_{i}^{2}},\\ \frac{\partial Q_{i}}{\partial E_{i}}=\frac{2X_{i}E_{i}-X_{i}V-R_{i}V\varphi_{i}}{R_{i}^{2}+X_{i}^{2}}. \end{array}$$

For power system applications, the line impedance is mainly inductive; that is, *R*_*i*_ ≈ 0. So (3) can be rewritten as follows:

(4) $$\begin{array}{c} \frac{\partial P_{i}}{\partial \varphi_{i}}=\frac{E_{i}V}{X_{i}},\;\;\frac{\partial P_{i}}{\partial E_{i}}=\frac{V\varphi_{i}}{X_{i}},\\ \frac{\partial Q_{i}}{\partial \varphi_{i}}=\frac{E_{i}V\varphi_{i}}{X_{i}},\;\;\frac{\partial Q_{i}}{\partial E_{i}}=\frac{2E_{i}-V}{X_{i}}. \end{array}$$

Considering that the power angle *φ*_*i*_ is much smaller than PCC voltage *V*, (∂*P*_*i*_)/(∂*φ*_*i*_)≫(∂*P*_*i*_)/(∂*E*_*i*_), and (∂*Q*_*i*_)/(∂*E*_*i*_)≫(∂*Q*_*i*_)/(∂*φ*_*i*_). In other words, the active power *P*_*i*_ is more dependent on the power angle (frequency) variation, while the reactive power *Q*_*i*_ is more sensitive to the output voltage magnitude variation. That is why *P*-*F* and *Q*-*V* droop control schemes are widely used in power systems:

(5) $$\begin{array}{c} \omega_{i}=\omega_{i}^{*}+m_{i}\left(P_{i}^{*}-P_{i}\right), \end{array}$$

(6) $$\begin{array}{c} E_{i}=E_{i}^{*}+n_{i}\left(Q_{i}^{*}-Q_{i}\right). \end{array}$$

### 2.2. Proposed Droop Theory

The conventional droop control works well under the assumption that the line impedance is mainly inductive. However, it is not always the case, especially in low voltage microgrid, where the *R*/*X* ratio of the transmission line is relatively high. In this case, the conventional power droop control might suffer from the poor power decoupling and power sharing. Therefore, many interesting solutions have been presented for alleviating the abovementioned problems at the expense of complicated algorithms.

On the other hand, let us recall the conventional droop control to find out the inherent reason for the poor power sharing. Substituting (1) into (5) and (6), the transfer function of the power droop control can be obtained as

(7) Pi(s)=miXiEiVs(Ri2+Xi2)+miXiEiVPi∗(s)    +XiEiVs(Ri2+Xi2)+miXiEiVωi∗(s)    +(RiEi−RiEiV)ss(Ri2+Xi2)+miXiEiV,Qi(s)=niXi(Ei−V)Ri2+Xi2+niXi(Ei−V)Qi∗(s)    +Xi(Ei−V)Ri2+Xi2+niXi(Ei−V)Ei∗(s)    +RiEiVφiRi2+Xi2+niXi(Ei−V).

For three-phase balanced ac system, the steady-state power is constant; that is, *s* = *jω* = 0. Therefore, (7) can be simplified as

(8) $$\begin{array}{c} P_{i}=P_{i}^{*}+\frac{1}{m_{i}}\omega_{i}^{*}, \end{array}$$

(9) Qi(s)=ni((Ri2+Xi2)/(Xi(Ei−V)))+niQi∗(s) +1((Ri2+Xi2)/(Xi(Ei−V)))+niEi∗(s) +RiEiVφiRi2+Xi2+niXi(Ei−V).

Equation (8) indicates that the active power of *i*th inverter is dependent on the power reference *P*_i_*, angular frequency reference *ω*_i_*, and droop coefficient *m*_*i*_. An accurate active power sharing can be easily achieved on the condition that the parameters of *P*_i_*, *ω*_i_*, and *m*_*i*_ are equally set, respectively. For example, the accurate sharing of active power among two inverters can be achieved on the condition that *P*_1_* = *P*_2_*, *ω*_1_* = *ω*_2_*, and *m*_1_ = *m*_2_, which can be well defined in practice. It can also be easily understood from (5) that *P*_1_ = *P*_2_ is valid under the aforementioned three conditions along with *ω*_1_ = *ω*_2_, which is always true because the frequency remains the same at any node along the transmission line.

On the other hand, (9) implies that the reactive power of *i*th inverter is dependent on not only the well-defined parameters of *Q*_i_*, *E*_i_*, and *n*_*i*_ but also the PCC voltage *V* and the line impedance *R*_*i*_ + *jX*_*i*_. That is why the reactive power sharing is difficult to achieve in practice, which is mainly due to unequal line impedances.

**Figure 2:**
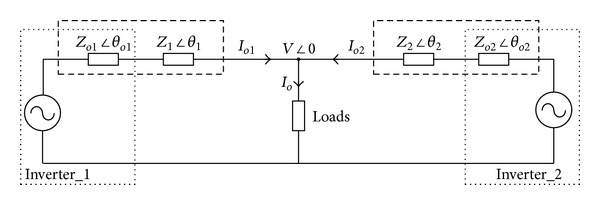
Simplified diagram of inverter-based microgrid.

In order to solve the above-mentioned problem, a new droop control based on impedance match concept is proposed in this paper. As stated earlier, the *overall impedance* in the droop control of inverters depends on not only the line impedance *Z*_*oi*_ *∠θ*_*o*__*i*_ but also the inverter output impedance *Z*_*i*_ *∠θ*_*i*_, as illustrated in Figure 2.

Note that the inaccuracy of reactive power sharing is mainly caused by the unequal impedance. Therefore, the inverter output impedance should be carefully designed to ensure that the overall impedance of each inverter is equal; that is to say, *Z*_*o*1_ *∠θ*_*o*__1_ + *Z*_1_ *∠θ*_1_ = *Z*_*o*2_ *∠θ*_*o*__2_ + *Z*_2_ *∠θ*_2_. In this way, the reactive power will be accurately shared. It can also be easily understood from (9) and Figure 2. With overall impedance, Q*, E* and *n* of each inverter equal, *Q*_1_ = *Q*_2_ will be valid.

## 3. System Loop Control Design

**Figure 3:**
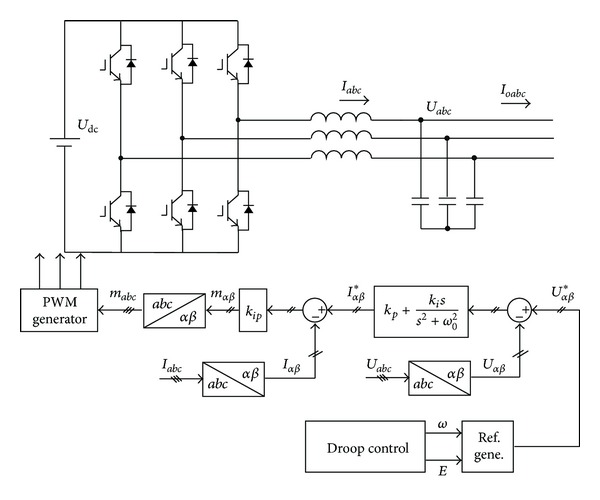
Block diagram of system loop control.

In this section, the design guideline of the system loop control is presented to achieve the high performance in both steady state and transient state.  Figure 3 illustrates the block diagram of the system loop control, which consists of inner current/voltage control loop and external power droop control loop.

### 3.1. Current Loop Design

**Figure 4:**
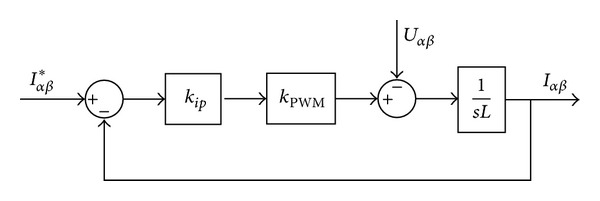
Block diagram of the inner current control loop.

The block diagram of the current control loop is depicted in Figure 4, where *L* is filter inductor and *K*_PWM_ is the PWM gain.

The closed-loop transfer function of the current control loop can be derived from Figure 4 as follows:

(10) $$\begin{array}{c} T_{i}\left(s\right)=\frac{I_{\alpha \beta }\left(s\right)}{I_{\alpha \beta }^{*}\left(s\right)}=\frac{k_{ip}K_{\text{PWM}}}{Ls+k_{ip}K_{\text{PWM}}}. \end{array}$$

**Figure 5:**
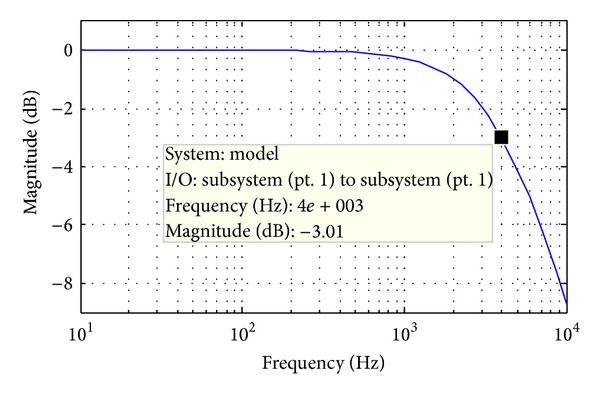
Bode diagram of current control loop.

In general, the bandwidth of the current control loop should be high enough to achieve the fast dynamic response [13, 14]. So the bandwidth (4 kHz, as shown in Figure 5) is selected by one-fifth of the switching frequency (20 kHz), which means *k*_*pi*_ ≈ 0.6.

### 3.2. Voltage Loop Design

Figure 5 shows the block diagram of the voltage control loop, where *T*_*i*_(*s*) is the transfer function of the current control loop and *C* is the filter capacitor.

**Figure 6:**
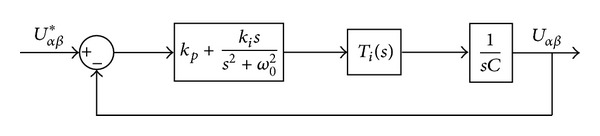
Block diagram of the voltage control loop.

The closed-loop transfer function of the voltage control loop can be derived from Figure 6 as follows:

(11) $$\begin{array}{c} T_{v}\left(s\right)=\frac{\left(k_{ip}K_{\text{PWM}}\right)\left(k_{p}s^{2}+k_{i}s+k_{p}\omega_{o}^{2}\right)}{LCs^{4}+k_{ip}CK_{\text{PWM}}s^{3}+D_{2}s^{2}+D_{1}s+D_{0}}, \end{array}$$

where

(12) $$\begin{array}{c} D_{2}=k_{p}k_{ip}K_{\text{PWM}}+LC\omega_{o}^{2},\\ D_{1}=k_{ip}K_{\text{PWM}}\left(C\omega_{o}^{2}+k_{i}\right),\\ D_{0}=k_{p}k_{ip}K_{\text{PWM}}\omega_{o}^{2}. \end{array}$$

**Figure 7:**
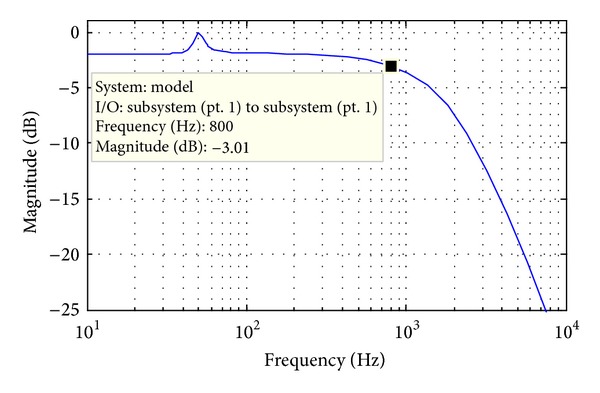
Bode diagram of voltage control loop.

The bandwidth of the voltage control loop should be carefully designed to avoid the dynamic interaction with current control loop. In this paper, the bandwidth (800 Hz, as shown in Figure 7) is selected by one-fifth of the current control loop (4 kHz), which means *k*_*p*_ ≈ 0.05 and *k*_*i*_ ≈ 3.2.

### 3.3. Output Impedance Design

As stated earlier, the output impedance has a potential impact on the accuracy of reactive power sharing. Therefore, this section will provide the analysis and design of the output impedance.

**Figure 8:**
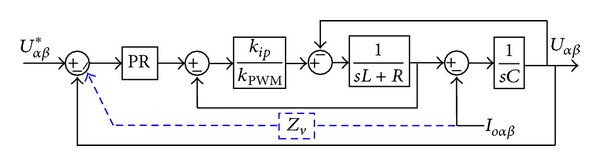
Bode diagram of voltage/current control loop.

The overall block diagram of the voltage/current control loop is illustrated in Figure 8, where *R* is the equivalent series resistor of the inductor. *Z*_*v*_(*s*) represents the virtual impedance.

The overall output impedance can be derived from Figure 8 as

(13) Zo(s)=−Uαβ(s)Iαβ(s)=−((s2+ωo2)(Ls+R)+Zv(s)kipKPWMD(s)) ×([LCs2+(kipKPWM+R)Cs+1]   ×(s2+ωo2)+kipKPWMD(s))−1,

where *D*(*s*) = *k*_*p*_ *s* ^2^ + *k*_*i*_ *s* + *k*_*p*_ *ω*_*o*_^2^.

When the virtual impedance is disabled (*Z*_*v*_ = 0), the output impedance can be simplified as follows:

(14) Zo(s)=−((s2+ωo2)(Ls+R)) ×([LCs2+(kipKPWM+R)Cs+1]   ×(s2+ωo2)+kipKPWMD(s))−1.

**Figure 9:**
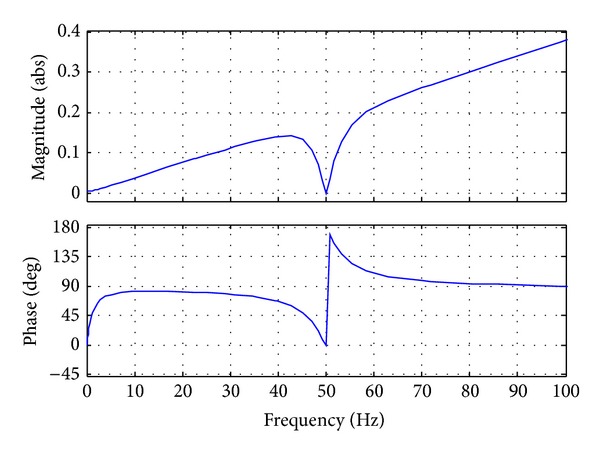
Bode diagram of output impedance (*Z*_*v*_ = 0).

From (14), it can be concluded that the output impedance at the fundamental frequency is *zero*, as shown in Figure 9. And it is in contrast with [7], where the PI control coefficients and filter parameters have a significant effect on the output impedance.

On the other hand, when the virtual impedance is enabled (*Z*_*v*_ ≠ 0), the overall output impedance at the fundamental frequency can be derived from (13) as follows:

(15) $$\begin{array}{c} Z_{o}\left(s\right)=-Z_{v}\left(s\right). \end{array}$$

The virtual impedance should be designed to be inductive or resistive according to the impedance match concept, as discussed in the previous section.

## 4. Performance Evaluation

In order to verify the effectiveness of the proposed control scheme, the time-domain simulations in MATLAB/Simulink are carried out according to Figure 1. The system parameters are given as follows. The dc and ac bus voltages are 250 V and 120 V/50 Hz, respectively. The LC filter parameters are 3 mH and 9.9 uF. The switching frequency is 20 kHz. The control parameters are given in Section 3. The line impedances are set to be unequal (*Z*_1_ = 0.5 + *j*0.314 and *Z*_2_ = 1 + *j*0.628) to evaluate the conventional and proposed droop control schemes. Before 1.5 s, the conventional droop control is used. After 1.5 s, the proposed droop control is enabled. Simulation results are provided as follows.

**Figure 10:**
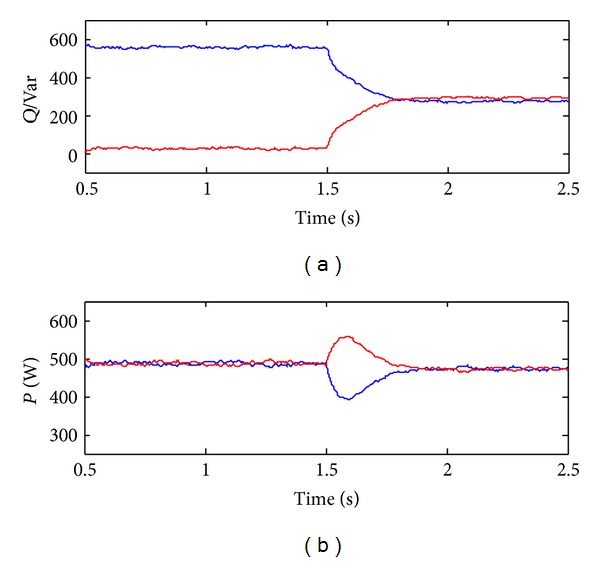
Simulation results of power sharing with conventional and proposed control. (a) Reactive power. (b) Active power.

Figure 10(a) illustrates the simulation results of the reactive power sharing. In agreement with the theoretical analysis in Section 2, the conventional droop control has the poor reactive power sharing due to unequal line impedance. On the other hand, the proposed droop control achieves the accurate reactive power sharing by tuning the virtual impedance with the impedance match concept.

Note that the active power sharing is satisfactory, as shown in Figure 10(b), which indicates that the accuracy of active power sharing in steady state is not sensitive to the unequal line impedance with both conventional and proposed droop control schemes.

**Figure 11:**
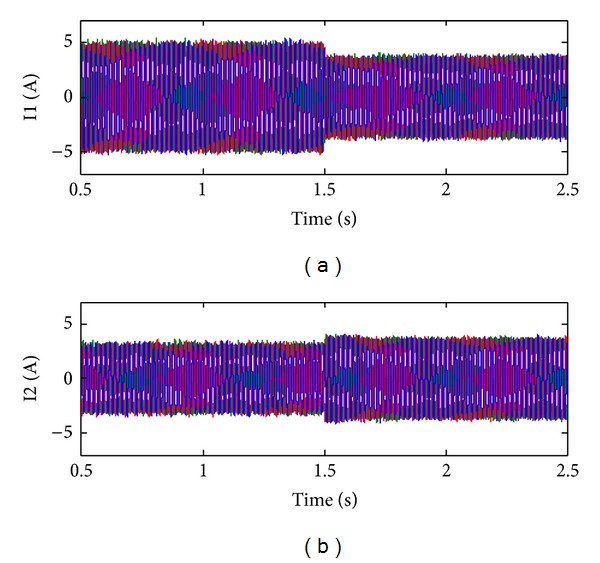
Simulation results of current sharing with conventional and proposed control. (a) Inverter_1. (b) Inverter_2.

Figure 11 shows the simulation results of the current sharing. The relatively higher current peak of inverter_1 than that of inverter_2 with the conventional droop control can be observed, which is mainly due to the unequal line impedance. After 1.5 s, the proposed droop control is activated. It is clear that the best current sharing is achieved as expected.

It should be noted that there is a trade-off between power sharing and line/feeder losses in case of unequal line/feeder impedances. The losses can be decreased if the load power is supplied mostly by the closer inverter (smaller feeder impedance). Further consideration is that the line/feeder impedance estimation should be required for the impedance match concept in the mesh microgrid, which would be reported in a future paper.

## 5. Conclusion

This paper has presented a comprehensive analysis and design of power sharing control for the inverter-dominated microgrid. From the theoretical analysis and simulation results, several conclusions can be reached as follow.

1. Conventional droop control can achieve the accurate active power sharing, even in the presence of high *R*/*X* ratio and unequal line impedance.
2. Conventional droop control fails to get better reactive power sharing, especially in case of unequal line impedance.
3. Reactive power sharing mainly depends on not only the line impedance but also the output impedance of each inverter.
4. Accurate reactive power sharing can be achieved on the condition that the *overall impedance* is matched for each inverter in microgrid, even in the presence of high *R*/*X* ratio and unequal line impedance.
